# An Exfoliated Graphite-Based Bisphenol A Electrochemical Sensor

**DOI:** 10.3390/s120911601

**Published:** 2012-08-27

**Authors:** Thabile Ndlovu, Omotayo A. Arotiba, Srinivasan Sampath, Rui W. Krause, Bhekie B. Mamba

**Affiliations:** 1 Department of Applied Chemistry, University of Johannesburg, P.O. Box 17011, Doornfontein 2028, Johannesburg, South Africa; E-Mails: thabilenbk@gmail.com (T.N.); sampath2562@gmail.com (S.S.); r.krause@ru.ac.za (R.W.K.); bmamba@uj.ac.za (B.B.M.); 2 Indian Institute of Science, Department of Inorganic and Physical Chemistry, Bangalore 560012, India

**Keywords:** exfoliated graphite electrode, bisphenol A, phenol, electrode fouling, pollutant

## Abstract

The use of an exfoliated graphite (EG) electrode in the square wave voltammetric detection of bisphenol A (a model phenolic pollutant) in water, whereby the phenolic electrode fouling challenge is mitigated, is described. The oxidation peak of BPA was observed at about 0.45 V in phosphate buffer solution at pH 10. The current response exhibited a linear relationship with the concentration over a range from 1.56 μM–50 μM. The detection limit was calculated to be 0.76 μM. The EG electrode surface was renewed after each measurement with excellent reproducibility. A real sample application was also investigated.

## 1. Introduction

A huge number of industrial processes involve the use and/or production of phenolic compounds. For example, bisphenol A (BPA) is widely used as a monomer and additive in the production of plastics, resins and coatings which are extensively used in food-packaging and dentistry [1]. Most phenolic compounds are toxic and hence constitute pollutants in water, food, soil and the environment at large. As regards toxicity, BPA has been identified recently as an emerging environmental pollutant owing to its endocrine disrupting activities [2,3]. Endocrine disrupting compounds are organic compounds that can cause negative effects on the endocrine systems of humans and wildlife. The release of BPA into the environment can be through leakage from plastic packaging into water or food. This leakage is caused by heat and acidic or basic conditions which accelerate the hydrolysis of the ester bonds linking BPA monomers [1].

The economic importance of phenols has resulted in the development of many methods for their detection and quantification [4]. However, owing to some advantages such as low cost and miniaturisability, phenol sensing using electrochemical transducers has been widely studied [5,6]. The electroanalysis of phenols and substituted phenols is possible due to the oxidation of the electroactive phenolic group [7,8]. The electrochemical detection of BPA on different modified electrodes has been reported in the literature. For example, Li *et al.* used a glassy carbon electrode (GCE) modified with carboxylated multiwalled carbon nanotubes (MWCNTs) to obtain a detection limit of 5 nM [9]. A similar work using gold nanoparticle (AuNPs) impregnated MWCNTs was reported earlier by Tu *et al.* [10]. In another study, BPA has been detected using a pencil graphite electrode modified with polyaniline nanorods and MWCNTs [11]. These reports were all based on some modified form of a carbon based electrode.

The major problem with electrochemical detection of phenols (including BPA) is electrode fouling which results from phenol polymerisation [5,6,9]. The most common way of dealing with electrode fouling problems is to modify the electrode surface or use composite materials containing nanoparticles with electrocatalytic behaviour [6,9]. However, fouling still occurs and this affects the sensitivity, reusability and reproducibility of the electrode. When using modified electrodes for the detection of phenolic compounds, the electrode is usually polished and re-modified again for every new measurement. This modification step (e.g., drop-dry or dip-coating methods) usually increases the analysis time [10,11]. Hence in this study, we present a low cost bare exfoliated graphite (EG) electrode, possessing a surface that can be renewed with good reproducibility, as a way of tackling the problem of fouling in BPA detection.

Exfoliated graphite is a low density material produced from graphite. It has good electrochemical properties and can be easily compressed for electrode fabrication [12]. Although EG electrodes have been available for some time now, there are currently no reports on the use of these cheap electrodes for the electroanalysis of water pollutants–an attempt which we have made in this report.

## 2. Experimental Section

### 2.1. Materials

Natural graphite and bisphenol A (BPA) were obtained from Sigma Aldrich while KH_2_PO_4_, K_2_HPO_4_, CH_3_CN, H_3_PO_4_ and NaOH were purchased from Merck Chemicals. A 1 mM stock solution of BPA was prepared by dissolving the right amount of BPA in a minimum amount of acetonitrile and diluting with 0.1 M phosphate buffer solution which was kept in the fridge. Phosphate buffer solution was used as the supporting electrolyte for all electrochemical experiments. All electrochemical measurements were done on an AutolabPGSTAT 302N unit using a three-electrode configuration. Working electrode, counter electrode and reference electrode were an EG (5 mm diameter), platinum wire and Ag/AgCl (3 M Cl^−^) respectively. All solutions were de-aerated by purging with argon gas for 10 min and maintaining an argon atmosphere throughout the experiments.

### 2.2. Electrode Fabrication

EG was prepared as described before [13,14]. Briefly, natural graphite was intercalated with bisulphate ions. This resulted in graphite intercalated compounds (GIC) which on exposure to thermal shock at 800 °C yielded EG. The EG particles were restacked without any binder to form pellets/sheets by compressing approximately a 1 g weight at a pressure of 58 kPa for 6 hours. Electrodes were then fabricated from these pellets using a glass rod, copper wire, conduction silver paint and a piece of the recompressed EG [13]. A puncher was used to cut the EG pellet into a 5 mm diameter circle. One end of the copper wire was coiled and glued to one side of the EG pellet using a conducting silver paint. This piece was inserted into a glass tube and sealed (at the EG end) with an insulator (Araldite epoxy resin) leaving the other basal plane side exposed as the electrode surface. The Araldite was left to completely dry overnight. The electrode was characterized using cyclic voltammetry (CV) in the presence of K_4_[Fe(CN)_6_]/K_3_[Fe(CN)_6_] ([Fe(CN)_6_]^3−/4−^) redox probe.

### 2.3. Electrochemical Detection of Bisphenol A

The EG electrode was used to record CV and square wave voltammetry (SWV) of 25.0 μM BPA. All BPA experiments were done in PBS after optimizing the pH and pre-concentration time. Different concentrations (1.56 μM to 50.0 μM) of BPA were prepared and their SWV were used to plot the calibration curve and calculate the detection limit. All SWV experiments were done at room temperature at amplitude 50 mV and a frequency of 25 Hz. For real sample analysis, BPA was extracted from three samples of plastic containers using a method reported by Tu *et al.* [10]. Briefly, the plastic bottles were cut into small pieces and washed with distilled water. The pieces (4 g) were placed in a 100 mL flask and 50 mL distilled water was added. This was sealed using parafilm, ultrasonicated for 30 minutes and kept overnight at 70 °C for 48 hours. The filtrate was diluted to 100 mL in a volumetric flask. The container samples will be referred to as B1, B2 and B3.

## 3. Results and Discussion

### 3.1. Characterization of EG

**Figure 1.**
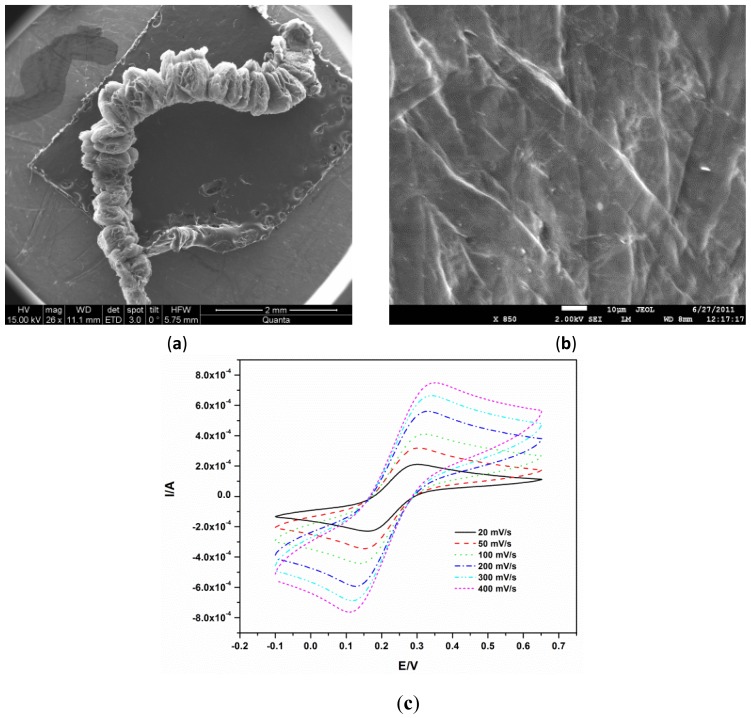
(**a**) SEM image EG; (**b**) SEM image of compressed EG and (**c**) Cyclic voltammetry of bare EG electrode in 5 mM [Fe(CN)_6_]^3−/4−^ (in 0.1 M KCl) at different scan rates.

The exfoliation of graphite is due to the temperature shock that leads to the vaporisation and violent expulsion of the bisulphate ions accommodated between the graphite layers [12]. This expulsion forces the graphite layers to separate. The resulting exfoliated graphite is a puffed material with a density of 0.0068 g·mL^−1^ as shown in a scanning electron micrograph Figure 1(a). Scanning electron microscopy also showed the disappearance of the typical accordion-like structure of the expanded graphite Figure 1(b) after compression, signifying the interlocking of the layers as previously reported [14]. The EG electrode was first characterized electrochemically using [Fe(CN)_6_]^3−/4−^. The characteristic diffusion controlled, quasi-reversible kinetics of [Fe(CN)_6_]^3−/4−^ Figure 1(c) were observed, illustrating the usability of EG as an electrode and validating the earlier reports that EG can be used as an electrode material [12,15].

### 3.2. Electrochemical Detection of BPA

**Figure 2.**
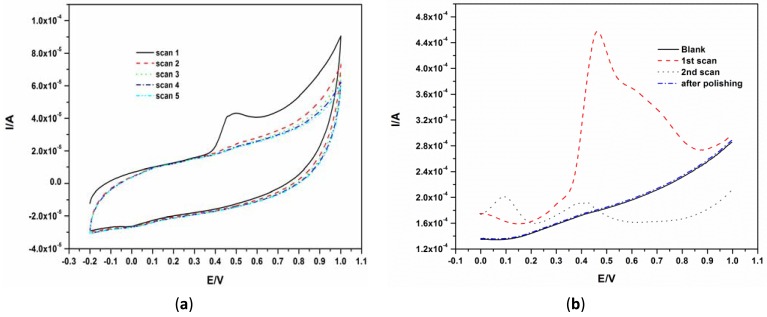
(**a**) CVs of 40 μM BPA in 0.1 M phosphate buffer solution. The scan rate was 50 mV·s^−1^ and five scans were recorded; (**b**) SWV for 40 μM BPA in phosphate buffer solution at pH 10 at an amplitude of 50 mV and a frequency of 25 Hz. The ‘after polishing’ scan was recorded in blank phosphate buffer solution.

Since there are no reports on the behavior of phenols on the EG electrode surface, the electrochemical behavior of BPA was first investigated using CV and SWV by scanning between −0.2 V and 1.0 V for CV and between 0 V and 1.0 V for SWV. A well-defined irreversible oxidation peak was observed at about 0.45 V as shown in Figure 2(a). The marked reduction and disappearance of this peak at subsequent scans Figure 2(a,b) was caused by the well-known fouling effect caused by the phenol group owing to the formation of a polymeric film on the electrode surface [6,9,10,16]. The oxidation peak at about 100 mV in the second scan seen in Figure 2(b) is due to the oxidation of polymer by-products. The surface of the EG electrode was easily renewed by polishing the electrode surface using emery paper with a fine grid of 1,600 as seen in the ‘after polishing scan’ of Figure 2(b).

**Scheme 1.**
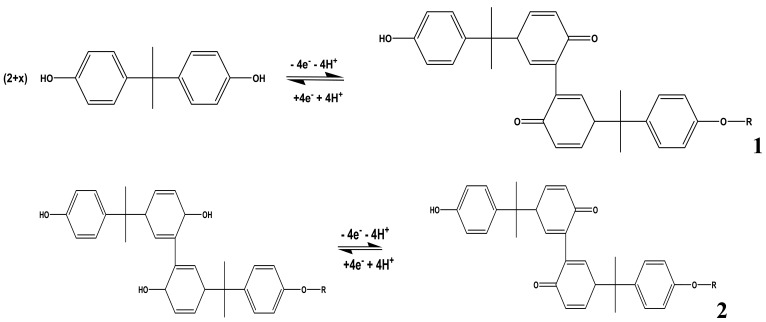
Mechanism for BPA electro-oxidation on solid electrode surface.

The mechanism for the electro-oxidation of phenols has been documented. For BPA, Zhang *et al.* proposed the mechanism shown in Scheme 1 [17]. They reported that the deposition process of BPA is controlled by the oxidation of BPA on the electrode surface. The initial stage in the oxidation of phenols after 4-electron transfer leads to the formation of quinones [16,18]. The subsequent peaks which develop after the first scan were ascribed to the redox reactions of the deposited coating or polymer as observed in 2nd scan of Figure 2(b). The electro-deposited products of BPA were proposed to contain several oxidizing centers which are known to contain *o*-quinone or *p*-quinone via a four electron and four-proton process [17].

### 3.3. pH and Preconcentration Time Optimization

**Figure 3.**
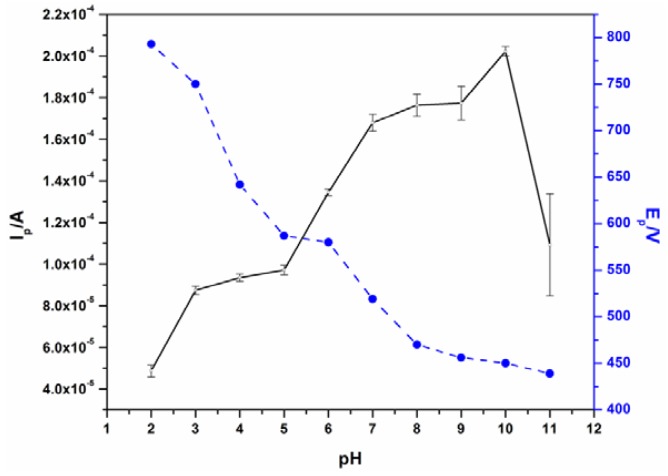
Effect of pH on the peak current (solid line) and peak potential (dotted line) on 30 μM BPA using SWV. The pre-concentration time for all pH optimisation measurements was 5 minutes.

The effect of pH on the peak current (I_p_) and peak potential (E_p_) was investigated between pH 2 and 11 as shown in Figure 3. At pH of 10, the highest I_p_ and a low E_p_ were observed. Thus, pH 10 was chosen as the optimum pH for all BPA measurements using the bare EG electrode. The pre-concentration of BPA was done under open circuit while stirring. A pre-concentration time of 6 minutes after which no BPA current increase was observed was chosen as the optimum (see Supplementary Figure S1).

#### 3.3.1. Detection Limit and Reproducibility

**Figure 4.**
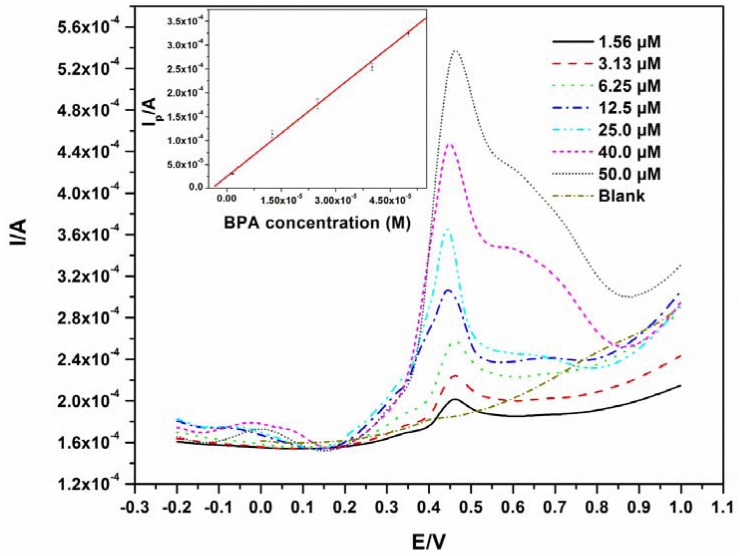
SWVs of different concentrations of BPA performed at an amplitude of 50 mV and a frequency of 25 Hz. Inset: linear variation of SWV currents with BPA concentration.

The detection limit was determined from a BPA calibration curve where different concentrations of BPA (1.56 μM–50.0 μM) were analyzed by pre-concentrating while stirring for 6 minutes at pH 10. A linear relationship (R = 0.993) between peak current and concentration was observed as shown in Figure 4, with a linear regression equation of *y* = 6.06*x* + 2.47 × 10^−5^.

The detection limit was calculated to be 0.76 μM, which is in the same range as a few other electrodes [17,19]. The ideal situation for the analysis of BPA and phenols in general, is to find a modifying agent that can significantly reduce electrode fouling. Going by this route, lower detection limits have indeed been reported [7,20,21], but the challenges of reproducibility and fouling still exist, especially when the modification chemistry is not easily repeatable. In the authors' opinion, for techniques involving interfacial electrode modification, fouling problems are not generally eliminated, especially after multiple scans. Furthermore, some modification protocols may be complex and difficult to reproduce. The use of EG electrode involves no modification and the fouling was eliminated by polishing in our case. The experiments were repeated four times with relative standard deviations of no more than 5% for all replicates. The detection limits as well as the modifiers used for BPA detection using other electrodes are listed in Table 1. The detection limits obtained from the modified electrodes are all slightly lower than that from the EG electrode. This does not imply that the EG cannot be used as its detection limit is low enough for the detection of BPA in plastic containers which usually have higher concentrations than the obtained detection limit [10,22]. Han *et al.* analysed some river samples using a GCE modified with nitrogen-doped graphene sheets and chitosan and found BPA concentrations above 1 μM [23]. This suggests practical applications for the unmodified EG electrode. This method can give reliable results in a time efficient manner as there are no time consuming modification steps.

**Figure 5.**
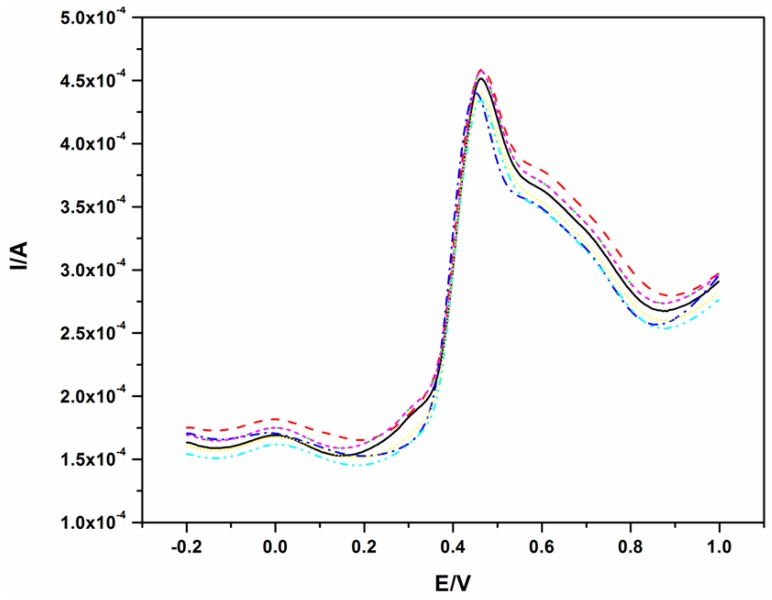
Seven SWVs of 40 μM BPA in phosphate buffer solution at pH 10.

To illustrate the reproducibility of this electrode, the SWV of 40.0 μM BPA was repeated seven times and the resulting voltammograms are shown in Figure 4. The oxidation of BPA, like on other electrodes, fouled the surface of the electrode as shown in Figure 2. Therefore, for all measurements in Figure 5, the electrode was polished and a background check (where a SWV was recorded in the absence of BPA before the next measurement) was performed. The polishing process did not affect the results as good reproducibility was obtained with a relative standard deviation of 3.4%.

There are currently no reports on the use of EG electrodes for electroanalysis of organic environmental pollutants. The first electrochemical studies on the EG electrode were reported by Frysz and Chung in 1997. They concluded that EG electrode offered better electron transfer rates and higher electrochemical area when compared to GCE [26]. Since then, somewhat surprisingly, this promising electrode has not been explored for the electroanalysis of water pollutants and other applications. The results obtained from this study suggest the possible use of EG electrodes in the monitoring of organic and inorganic water contaminants as an alternative electrode material.

Other phenolic compounds can act as interferents during BPA detection, thus the need to investigate the selectivity of this electrode. The oxidation peak currents of 25 μM BPA were recorded in the absence and presence of 2-nitrophenol, 3-nitrophenol, 4-nitrophenol and 4-chlorophenol. Peak current changes of less 9% were observed indicating an insignificant interference and thus a good selectivity. Such behaviour has also been observed on the detection of BPA using other electrodes [22–24]. This selectivity is mainly due to the fact that the oxidation potentials of these phenolic compounds are different from that of BPA. All the nitrophenols were oxidized at potentials less than 200 mV while 4-chlorophenol was oxidized at 740 mV on the EG electrode at pH 10. These values show a potential difference of more than 200 mV to that of BPA (oxidized at ca 440 mV). The difference in the observed oxidation potential could be due to the difference in the parent molecules which are all attached to different substituents.

#### 3.3.2. Detection of BPA in Bottles

The concentration of BPA in three commercial plastic samples used for food packaging was determined in order to ascertain the EG electrode's potential application to real samples. The BPA concentration was determined using standard addition and the concentrations of BPA found with the respective recovery in B1, B2 and B3 are listed in Table 2. B3 was labeled “BPA free” by the manufacturer and thus our result confirms the manufacturer's claim. The samples were spiked with certain amounts of BPA standard and the concentrations determined from triplicate measurements. The qualitative presence of BPA in the plastic material was confirmed using High Performance Liquid Chromatography (HPLC) with diode array detection (not shown). While bottle B1 and B2 were positive, B3 was negative corroborating our finding.

## 4. Conclusions

EG is a promising electrode material that has possible applications in environmental electroanalysis of phenolic pollutants. Polishing the fabricated EG electrode eliminated the effect of phenol fouling in the determination of BPA in water with good reproducibility. Since other phenolic pollutants exhibit this fouling behavior, the EG electrode lends itself to a versatile application in phenol sensing. Moreover, the electrode is low cost and easy to fabricate.

## Supplementary Material



## Figures and Tables

**Table 1.** Shows the detection limits of BPA obtained when using other electrodes by different researchers.

**Electrode**	**Modifier**	**Detection limit**	**Reference**
CPE	MCM-41 (mesoporous silica molecular sieves)	38 nM	[24]
Diamond electrode	Boron doped diamond electrode	0.21 μM	[25]
ITO electrode	Mediation of [Ru(bpy)_3_]^2+^	0.29 μM	[19]
Pencilgraphite	Polyanilinenanorods&MWCNT	10 nM	[11]
CPE	Cobaltphthalocyanine	10 nM	[6]
EG	None	0.76 μM	This work

**Table 2.** Determination of BPA in plastic containers.

**Sample**	**Added** **(μM)**	**Measured** **(μM)**	**% recovery**	**Calculated BPA concentration**
B 1	10.0	10.1	101 %	1.84 μM
	6.00	6.12	102 %	
B 2	20.0	20.8	104 %	1.92 μM
	2.00	1.96	98 %	
B 3	8.00	8.30	104 %	ND
	40.0	41.0	102 %	
