# Supplementary material for: An Exfoliated Graphite-Based Bisphenol A Electrochemical Sensor
Source: Sensors (Basel). 2012 Aug 27;12(9):11601–11. doi: 10.3390/s120911601 (PMC3478800; doi:10.3390/s120911601)
Supplement: Supplementary file 1 [file sensors-12-11601-s001.pdf]

OPEN ACCESS

sensors

ISSN 1424-8220
www.mdpi.com/journal/sensors

Supplementary Information

An Exfoliated Graphite-Based Bisphenol A Electrochemical Sensor. *Sensors* 2012, *12*, 11601-11611

Thabile Ndlovu [1], Omotayo A. Arotiba [1,*], Srinivasan Sampath [1,2], Rui W. Krause [1] and Bhekie B. Mamba [1]

[1] Department of Applied Chemistry, University of Johannesburg, P.O. Box 17011, Doornfontein 2028, Johannesburg, South Africa; E-Mails: thabilenbk@gmail.com (T.N.); sampath2562@gmail.com (S.S.); r.krause@ru.ac.za (R.W.K.); bmamba@uj.ac.za (B.B.M.)

[2] Indian Institute of Science, Department of Inorganic and Physical Chemistry, Bangalore 560012, India

* Author to whom correspondence should be addressed; E-Mail: oarotiba@uj.ac.za; Tel.: +2-711-559-6200, Fax: +2-711-559-6425.

Figure S1. Effect of pre-concentration time on the peak current of 40 µM BPA solution using SWV. The pH of the phosphate buffer was 10.

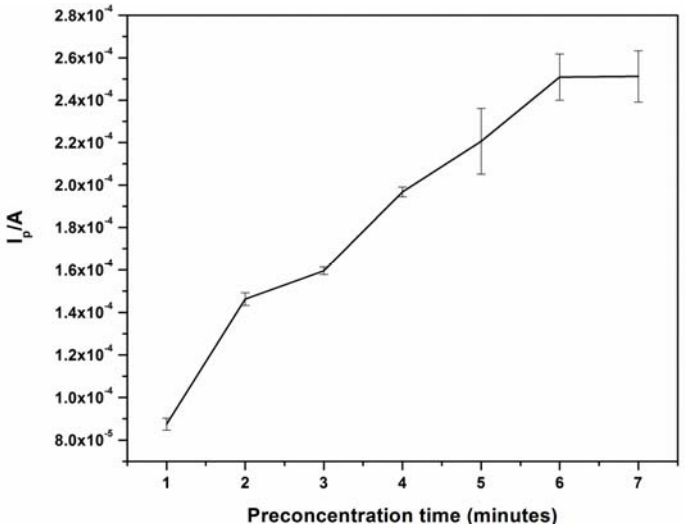

© 2012 by the authors; licensee MDPI, Basel, Switzerland. This article is an open access article distributed under the terms and conditions of the Creative Commons Attribution license (http://creativecommons.org/licenses/by/3.0/).
